# Boric Acid Suppresses Glioblastoma Cellular Survival by Regulating Ferroptosis via SOX10/GPx4/ACSL4 Signalling and Iron Metabolism

**DOI:** 10.1111/jcmm.70529

**Published:** 2025-03-30

**Authors:** Guven Kilic, Ceyhan Hacioglu, Cengiz Tuncer, Ezgi Kar, Fatih Kar, Ahmet Taskesen, Adem Kurtulus, Onder Ipek, Oben Devin Cetiner, Cigdem Erdin

**Affiliations:** ^1^ Department of Neurosurgery Düzce University, Faculty of Medicine Düzce Türkiye; ^2^ Department of Medical Biochemistry Düzce University, Faculty of Medicine Düzce Türkiye; ^3^ Department of Biochemistry Düzce University, Faculty of Pharmacy Düzce Türkiye; ^4^ Department of Nutrition and Dietetics Kütahya Health Sciences University, Faculty of Health Science Kütahya Türkiye; ^5^ Department of Medical Biochemistry Kütahya Health Sciences University, Faculty of Medicine Kütahya Türkiye

**Keywords:** boric acid, Ferroptosis, glioma, SOX

## Abstract

Ferroptosis, a distinct form of regulated cell death, plays a role in glioma pathogenesis. SRY‐box (SOX) transcription factors are key regulators of cancer progression. In this study, we investigated the role of SOX10 in ferroptosis induction in U87 cells following boric acid treatment. First, the cytotoxic effects of boric acid on HMC3 and U87 cells were assessed using CCK8 and BrdU incorporation assays. Subsequently, SOX10, GPX4, ACSL4, GSH, MDA, total ROS, Fe^2+^, and TFR levels were analysed using ELISA, Western blot, and RT‐PCR techniques. Additionally, DAPI staining was performed to evaluate nuclear abnormalities. According to the CCK8 analysis, the IC50 value for boric acid was determined to be 3.12 mM for HMC3 cells and 532 μM for U87 cells, a finding further supported by BrdU incorporation analysis, which indicated that U87 cells were more sensitive to boric acid. Western blot and RT‐PCR analyses revealed that SOX10 expression was significantly higher in U87 cells compared to HMC3 cells. Boric acid treatment led to a reduction in GSH, GPX4, and SOX10 levels in U87 cells, while inducing an increase in MDA, total ROS, ACSL4, Fe^2+^, and TFR levels. Moreover, microscopic analysis demonstrated that boric acid treatment induced both morphological and nuclear abnormalities in U87 cells. In conclusion, our findings demonstrate that SOX10 is involved in the ferroptosis signalling pathway and that boric acid effectively suppresses U87 cell viability by targeting the SOX10/GPX4/ACSL4 axis.

## 1 Introduction

Gliomas, which originate from glial cells, represent some of the most prevalent and lethal tumours of the central nervous system [1]. Glioblastoma, classified as a grade IV diffuse glioma, is the most common and aggressive form of primary brain tumour in adults [2]. This malignancy is associated with a median survival of approximately 1 year and a 5‐year survival rate of less than 5% [3]. Standard treatment protocols typically involve the surgical resection of the tumour, followed by a combined regimen of radiotherapy and temozolomide‐based chemotherapy [4]. Despite these interventions, current treatment approaches are largely ineffective, as patients almost invariably experience recurrence, often with increased aggressiveness. In recent years, numerous studies have expanded our understanding of the genetic and epigenetic landscape of glioblastoma [5]. These investigations have identified a wide range of genetic mutations and molecular signalling alterations that contribute to the disease's pathogenesis, highlighting the significant intratumoural heterogeneity characteristic of glioblastoma [6]. This pronounced heterogeneity poses substantial challenges to the development of targeted therapies. Consequently, there is an urgent need for innovative and effective treatment strategies.

The SOX (sex‐determining region Y (SRY)‐box) genes constitute a family of transcription factors characterised by a conserved high‐mobility group (HMG) DNA‐binding domain [7]. In humans, this family comprises 20 members, which are categorised into eight groups based on the sequence identity of their HMG domains [8]. This gene family has been associated with various diseases, and accumulating evidence indicates that several SOX members play a role in cancer development [9]. In tumour cells, SOX genes modulate immune cell infiltration through paracrine signalling, with reciprocal interactions also occurring [10]. Nearly all SOX genes, including SOX1, SOX2, SOX7, and SOX10, have been shown to influence glioma progression and exhibit oncogenic functions across multiple cancer types [11, 12, 13, 14]. These genes play a pivotal role in glioma by regulating processes such as the maintenance of stemness and the initiation of differentiation in glioma stem cells [15]. Within the SOX‐E subgroup, SOX8, SOX9, and SOX10 are potent modulators of both the peripheral and central nervous systems [15]. Among these, SOX10 has been identified as a critical member, contributing to the restoration of neurological functions [16]. Consequently, the SOX gene family is essential to glioma development. However, the specific roles of individual SOX genes in glioma, as well as their precise mechanisms of action, remain inadequately understood.

Ferroptosis is a recently identified form of cell death characterised by iron‐dependent accumulation of lipid hydroperoxides and subsequent membrane damage [17]. Dysregulation of iron homeostasis and lipid metabolism plays a critical role in the initiation of ferroptosis [18]. Sensitization to ferroptosis can be attained by diminishing the phosphorylation of acetyl‐CoA carboxylase, thereby facilitating the accumulation of lipids that are abundant in polyunsaturated fatty acids (PUFAs) [19]. This phenomenon is primarily mediated by lipid peroxidation, which is intensified by reactive oxygen species (ROS) and iron (Fe^2+^)‐catalysed reactions. Moreover, the modulation of acyl‐CoA synthetase long‐chain family member 4 (ACSL4) has been implicated in enhancing phospholipid oxidation, which in turn increases lipid peroxide production and activates ferroptotic signalling pathways [20]. In contrast, glutathione peroxidase 4 (GPx4) counteracts this process by utilising glutathione (GSH) to inhibit the activation of ferroptotic signalling and mitigate lipid peroxidation [21]. Ferroptosis has been associated with the effectiveness of various anticancer therapies, including radiotherapy, chemotherapy, targeted therapies and immunotherapy [21]. Metabolic reprogramming, a hallmark of glioblastoma, offers a promising therapeutic avenue to address drug resistance [22]. As previously noted, while the induction of ferroptosis holds promise for improving glioblastoma treatment outcomes, further investigation is essential to fully comprehend its effects and uncover the underlying mechanisms involved in glioblastoma progression.

Boron, a naturally occurring element, has attracted considerable attention due to its capacity to selectively accumulate in tumour tissues [23]. This property has spurred the development of boron‐based compounds for targeted cancer therapies. In addition, compounds such as boric acid and borax have been investigated as potential therapeutic agents in glioblastoma, with studies demonstrating their ability to reduce both antioxidant capacity and heat shock protein levels in glioblastoma cells [24]. Our previous research indicated that borax decreases cell viability and proliferation in U87 glioblastoma cells through the induction of ferroptosis [25]. Despite these promising findings, further investigation is required to elucidate the precise mechanisms underlying the anticancer effects of boron‐containing compounds.

The present study sought to investigate the mechanisms by which SOX10 influences tumour proliferation following boric acid treatment, as well as its role in ferroptosis and glioblastoma progression. Specifically, the study aimed to evaluate whether boric acid treatment in U87 glioblastoma cells could serve as a potential therapeutic strategy for glioblastoma by targeting the SOX10/GPx4/ACSL4 signalling axis.

## 2 Materials and Methods

### 2.1 Cell Culture

The human microglial clone 3 (HMC3) cells and human glioblastoma cells (U87) were obtained from the American Type Culture Collection (ATCC) and cultured in Dulbecco's Modified Eagle's Medium (DMEM) supplemented with 10% fetal bovine serum (FBS) and 2 mM glutamine. The cells were subsequently seeded into 75 cm^2^ culture flasks containing the prepared medium and maintained in an incubator at 37°C with 5% CO_2_.

### 2.2 Cell Viability and Proliferation Assays

Initially, HMC3 and U87 cells were seeded at a density of 2 × 10^5^ cells per well. Subsequently, the cells were treated with boric acid at concentrations ranging from 25 to 1.6 mM for 24 h. Cell viability was evaluated using the Cell Counting Kit 8 (CCK8, Cat. no. E‐CK‐A362) according to the manufacturer's instructions. In brief, the cells (2 × 10^5^) were incubated with the CCK‐8 solution at 37°C for the recommended duration, after which absorbance was measured at 450 nm. Following the 24 h treatment, cell proliferation was assessed using the 5‐bromo‐2′‐deoxyuridine (BrdU) incorporation assay (2750, Sigma‐Aldrich), again following the manufacturer's guidelines. This assay is based on the principle that BrdU is incorporated into the DNA of actively dividing cells during the S phase of the cell cycle, and it is subsequently detected using an anti‐BrdU antibody. The absorbance at 450 nm was recorded to quantify cell proliferation.

### 2.3 Morphology Analysis

To investigate structural modifications in the nuclei of U87 cells following boric acid treatment, nuclear morphology imaging was performed. A total of 2 × 10^5^ cells per well were exposed to boric acid for 24 h, after which they were stained with 4',6‐diamidino‐2‐phenylindole (DAPI; D8417, Sigma‐Aldrich). The staining process involved incubation in a DAPI solution for 30 min at 25°C under dark conditions. After incubation, the staining solution was discarded, and nuclear structures were visualised using fluorescence microscopy. Additionally, counterstaining was employed to improve the resolution of nuclear features.

### 2.4 Analysis of Fe^2+^ and Ferroptotic Biomarkers

Cells were initially seeded in 12‐well plates at a density of 2 × 10^5^ cells per well and incubated for 24 h to allow for adaptation to the culture medium. Subsequently, the cells were treated with boric acid for 48 h. After treatment, the cells were trypsinized and centrifuged at 1200 × g for 10 min at 4°C, and the resulting cell pellets were collected. The pellets were then resuspended in radioimmunoprecipitation assay (RIPA) buffer (R0278; Sigma‐Aldrich) supplemented with a protease inhibitor cocktail (P8340; Sigma‐Aldrich) and subjected to 20 s of sonication in an ultrasonic bath to disrupt cell membranes. The lysate was subsequently centrifuged at 10,000 × g for 15 min at 4°C to remove cell debris, and the supernatant, representing the cell lysate, was collected for further biochemical analyses.

The concentrations of Fe^2+^, malondialdehyde (MDA), glutathione (GSH), total reactive oxygen species (ROS), glutathione peroxidase 4 (GPx4), and acyl‐CoA synthetase long‐chain family member 4 (ACSL4) were quantified using commercial kits (MAK025, MAK085, MBS727656, BG‐HUM20964, MBS2000338, and MBS9331516, respectively) in accordance with the manufacturers' protocols. Prior to measurement, all reagents were equilibrated to room temperature. In brief, the cell lysates were dispensed into 96‐well plates pre‐coated with capture antibodies specific to the target proteins and incubated for the duration specified in the protocols. After the addition of the reaction solutions and the completion of washing steps, a stop solution was applied. Absorbance values were then measured at the designated wavelengths using a microplate reader, enabling the calculation of protein concentrations in each well.

### 2.5 Real‐Time Polymerase Chain Reaction and Western Blotting Analysis

Total RNA was extracted using the TRIzol reagent (Invitrogen, 12594025), and the RNA concentration and quality were assessed by measuring absorbance at 260 and 280 nm. Complementary DNA (cDNA) synthesis was performed using the SuperScript IV One‐Step Real‐Time Polymerase Chain Reaction (RT‐PCR) System (Thermo Fisher Scientific, 12594100) in accordance with the manufacturer's instructions. The resulting cDNA was diluted to a final volume of 50 μL for subsequent RT‐PCR analysis. RT‐PCR was performed using the SYBR Green reagent (Applied Biosystems). Relative gene expression levels were calculated using the 2^−ΔΔCT^ method, with GAPDH serving as the internal control for normalisation. The following primer sets were used for gene amplification: GPx4 forward 5'‐ACG GCT AGG ACT CCG CGA TC‐3', GPx4 reverse 5'‐AGC GTA GGG CTG GAC CGT CT‐3', ACSL4 forward 5'‐CGA CCT AAG GGA GTG ATG A‐3', ACSL4 reverse 5'‐CCT GCA GCC ATA GGT AAA GC‐3', SOX10 forward 5'‐GCT GCG GTA CCA GCC CTG TCA‐3', SOX10 reverse 5'‐CCA GCC GTG ACA GGA CAA GC‐3', GAPDH forward 5'‐ACC CAG AAG ACT GTG GAT GG‐3', GAPDH reverse 5'‐TTC TAG ACG GCA GGT CAG GT‐3'.

After 48 h of treatment with boric acid, cells were collected from the culture medium and lysed using RIPA buffer, followed by centrifugation to remove membrane debris. Protein concentrations were determined using the BCA Protein Assay Kit (71285, Millipore) according to the manufacturer's protocol. Subsequently, 20 μg of protein per well was separated on a 10% SDS‐PAGE gel and transferred onto a nitrocellulose membrane. The membranes were then blocked with 2% bovine serum albumin (BSA) for 1 h at room temperature and incubated overnight at 4°C with primary antibodies targeting the following proteins: GPx4 (1:1000, PA5‐102521), ACSL4 (1:2000, PA5‐27137), SOX10 (1:1000, PA5‐40697), transferrin receptor (TFR, 0.1 μg/mL, MA1‐40198), and GAPDH (1:2000, PA1‐987). After overnight incubation, the membranes were washed and incubated with HRP‐conjugated secondary antibodies for 1 h at room temperature. Protein bands were visualised using an appropriate detection system.

### 2.6 Statistical Analysis

Data analysis was performed using GraphPad Prism 8.0 software (GraphPad Software Inc., San Diego, CA). The results are expressed as the mean ± standard deviation (SD) from seven independent experiments. The Shapiro–Wilk normality test was utilised to determine whether the data conformed to a normal distribution, and the data were confirmed to satisfy the assumptions of normality and homogeneity of variance. Statistical differences between two groups were assessed using the Student's *t*‐test, while comparisons among multiple groups were conducted using either one‐way or two‐way analysis of variance (ANOVA), followed by Tukey's multiple comparison test. A *p*‐value of less than 0.05 was considered statistically significant.

## 3 Results

### 3.1 Boric Acid Affected Cell Viability and Proliferation in HMC3 and U87 Cells in a Cell Type‐Dependent Manner

**FIGURE 1:**
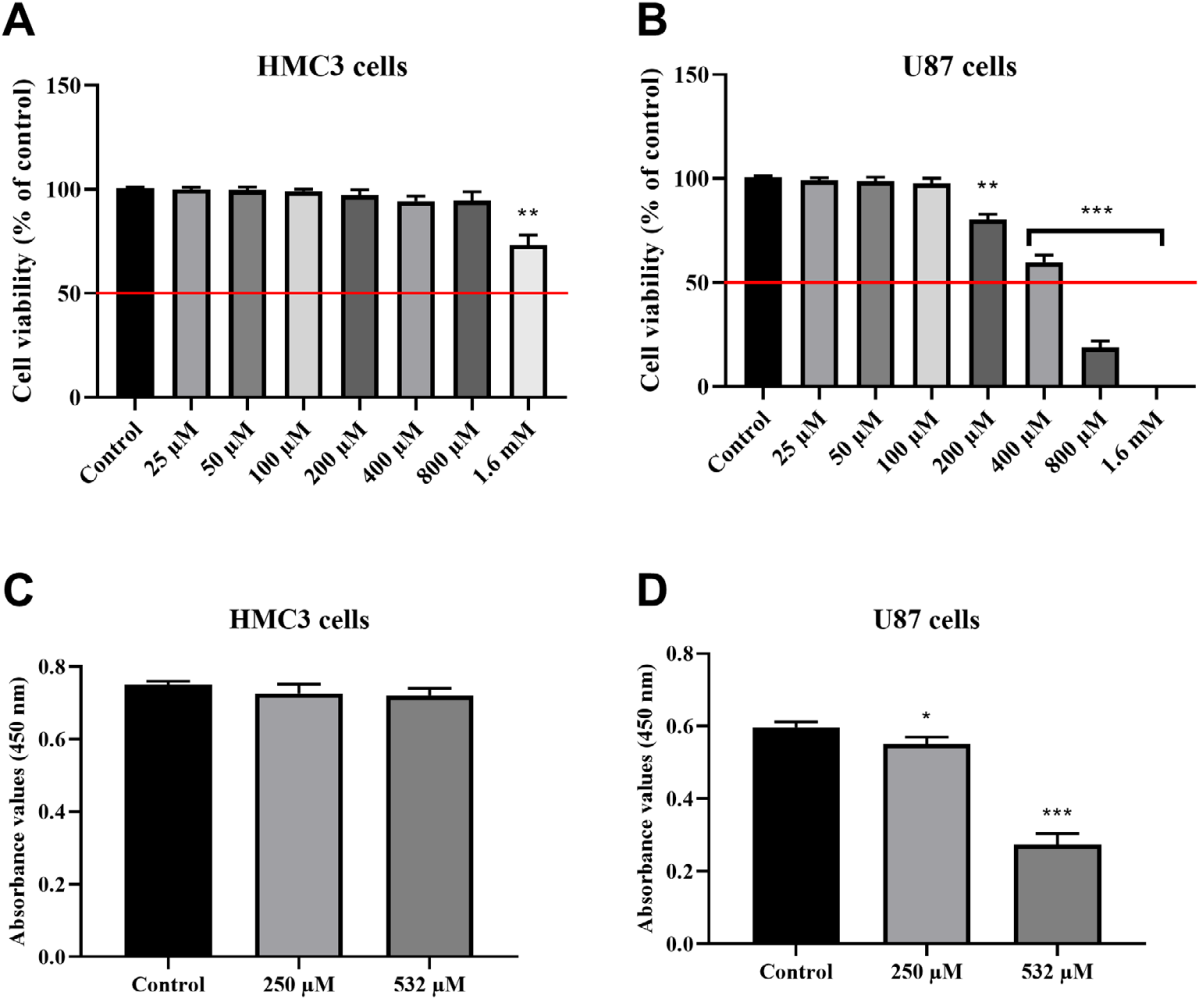
Inhibitory effects of boric acid on cellular viability and proliferation in HMC3 and U87 cells. (A) CCK8 analysis in HMC3 cells, (B) CCK8 analysis in U87 cells, (C) BrdU analysis in HMC3 cells; (D) BrdU analysis in U87 cells. *p < 0.05, **p < 0.001 and ***p < 0.0001 compared to control groups.

Boric acid treatment produced concentration‐dependent effects on cell viability and proliferation in both HMC3 and U87 cells (Figure 1). In HMC3 cells, exposure to boric acid concentrations ranging from 25 to 1.6 mM elicited noticeable changes in cell viability compared with the control group (Figure 1A). At concentrations up to 800 μM, a moderate yet statistically nonsignificant decrease in HMC3 cell viability was observed after 24 h of treatment (*p* > 0.05). However, treatment with 1.6 mM boric acid significantly reduced cell viability by 28.3% (*p* < 0.001 vs. control). In U87 cells, the CCK8 assay revealed no significant reduction in cell viability at boric acid concentrations between 0 and 100 μM (*p* > 0.05, Figure 1B). In contrast, 48 h treatments with 200, 400, and 800 μM boric acid resulted in marked decreases in cell viability, reducing it to 21.5%, 39.2%, and 80.6% of the control levels, respectively (*p* < 0.001 and *p* < 0.0001 vs. control). At the highest concentration of 1.6 mM, U87 cell viability was completely abolished. Based on the CCK8 data, the half‐maximal inhibitory concentration (IC50) of boric acid was calculated as 532 μM for U87 cells and 3.12 mM for HMC3 cells, indicating that HMC3 cells are more resistant to the cytotoxic effects of boric acid compared with U87 glioblastoma cells.

Furthermore, the BrdU assay demonstrated that treating HMC3 cells with 250 μM and 532 μM boric acid for 24 h did not significantly inhibit cell proliferation relative to the control group (*p* > 0.05, Figure 1C). Conversely, treatment of U87 cells with the same concentrations of boric acid resulted in a notable reduction in cell proliferation, with decreases of 18.3% and 56.5%, respectively, at 24 h (*p* < 0.05 and *p* < 0.0001 compared to the control, Figure 1D). These findings highlight the differential responses to boric acid treatment across various cell types. Specifically, boric acid exhibited weaker anti‐proliferative effects in HMC3 cells, which appeared less affected by the treatment. In contrast, U87 glioblastoma cells displayed heightened sensitivity to boric acid, demonstrating stronger anti‐proliferative effects. This variability underscores the importance of considering cell type‐specific responses when evaluating boric acid as a potential therapeutic agent.

### 3.2 Boric Acid Induced Abnormalities in Cell Morphology and Prooxidant/Oxidant Imbalance in U87 Cells

**FIGURE 2:**
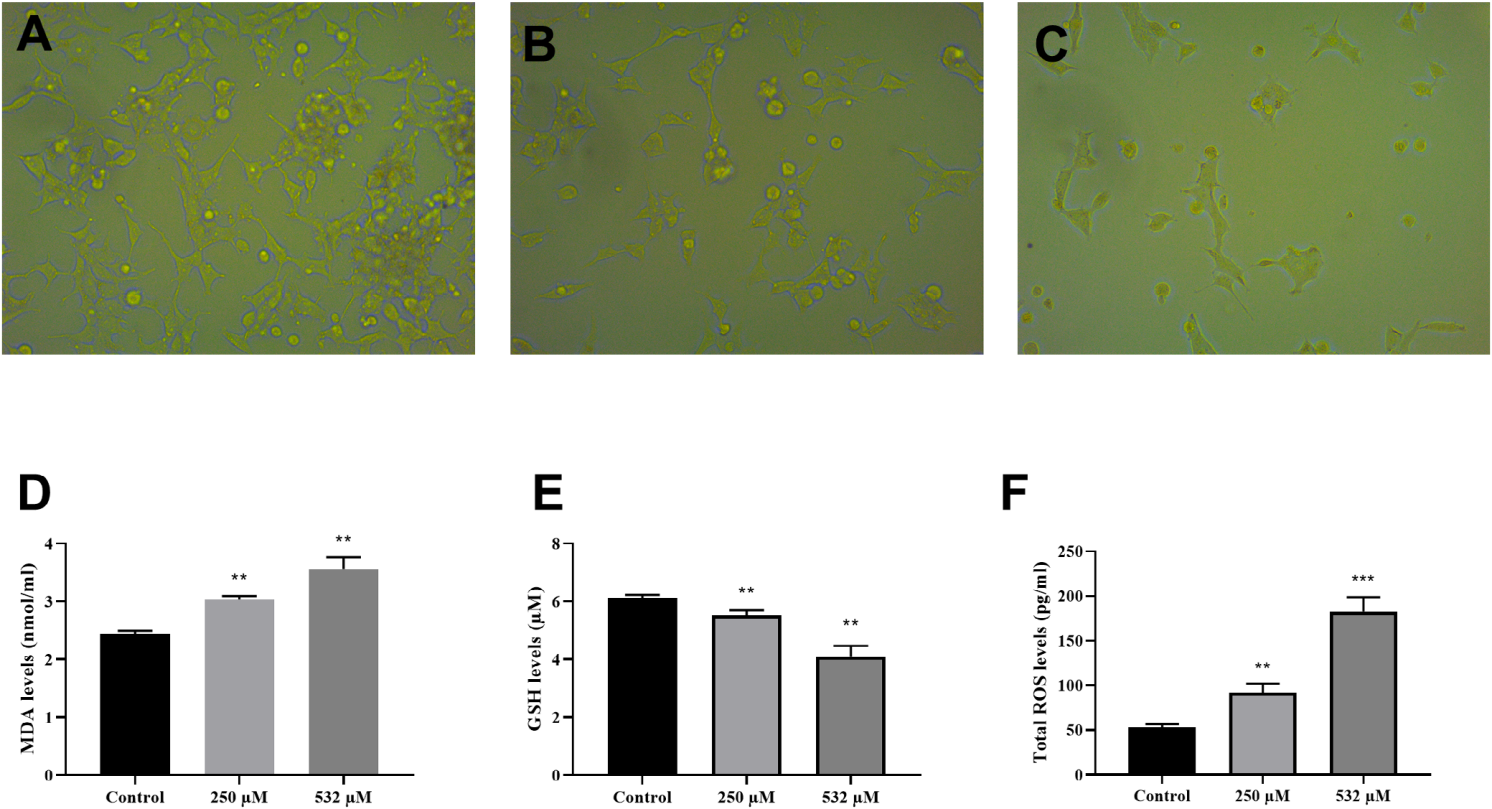
Effect of boric acid on cellular morphology, lipid peroxidation and prooxidant/oxidant balance in U87 cells. (A) Inverted microscope images of U87 cells in control group, (B) Inverted microscope images of U87 cells treated with 250 μM boric acid, (C) Inverted microscope images of U87 cells treated with 532 μM boric acid, (D) MDA results of boric acid treated U87 cells, (E) GSH results of boric acid treated U87 cells, (F) Total ROS results of boric acid treated U87 cells. Images in (A–C) were captured using a 20× objective lens. **p < 0.001 and ***p < 0.0001 compared to control groups.

Since CCK8 and BrdU analyses demonstrated that U87 cells were more sensitive to the cytotoxic effects of boric acid compared to HMC3 cells, subsequent analyses were focused on U87 cells. Observations under an inverted microscope revealed a concentration‐dependent reduction in cell numbers in boric acid‐treated U87 cells (Figure 2A–C). Additionally, cellular morphological abnormalities were noted, including reductions in cell size and significant decreases in cytoplasmic volume, which became more pronounced with increasing boric acid concentrations.

ELISA analyses further confirmed oxidation load‐related changes in boric acid‐treated U87 cells. Specifically, boric acid exposure led to a significant increase in the levels of the lipid peroxidation‐associated biomarker MDA (*p* < 0.0001, Figure 2D) and a concurrent decrease in GSH levels (*p* < 0.0001, Figure 2E). Moreover, these molecular alterations were accompanied by an increase in reactive oxygen species (ROS) levels in boric acid‐treated U87 cells (Figure 2F).

### 3.3 Boric Acid Regulates the SOX10/Ferroptosis Axis and Iron Metabolism

**FIGURE 3:**
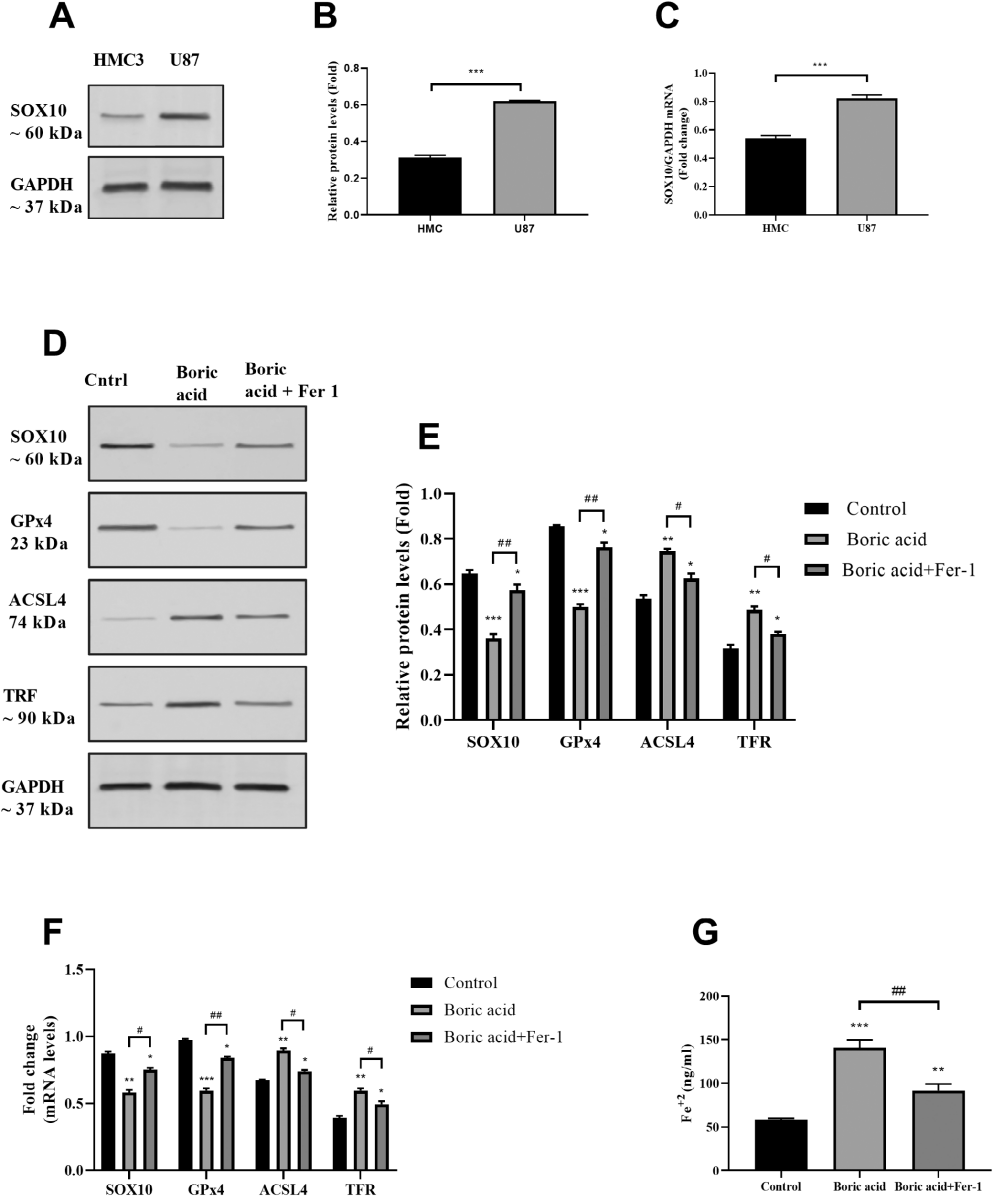
Effects of boric acid on SOX10/GPx4/ACSL4 signalling pathway. (A) Western blot analysis of SOX10 in HMC3 and U87 cells, (B) SOX10 protein levels in HMC3 and U87 cells, (C) SOX10 mRNA levels in HMC3 and U87 cells, (D) Western blot analysis of SOX10, GPx4, ACSL4 and TFR in U87 cells treated with boric acid and boric acid+Fer‐1, (E) SOX10, GPx4, ACSL4 and TFR protein levels in U87 cells treated with boric acid and boric acid+Fer‐1, (F) SOX10, GPx4, ACSL4 and TFR mRNA levels in U87 cells treated with boric acid and boric acid+Fer‐1, (G) Fe^+2^ levels in U87 cells treated with boric acid and boric acid+Fer‐1. *p < 0.05, **p < 0.001 and ***p < 0.0001 compared to control groups. # *p* < 0.05 and ## *p* < 0.001 compared to boric acid treated group.

Initially, an analysis was conducted to examine the differences in SOX10 expression between HMC3 and U87 cell lines (Figure 3). Western blot and RT‐PCR results revealed that SOX10 protein and mRNA levels were relatively higher in U87 cells compared to HMC3 cells (Figure 3A–C). Subsequently, the relationship between boric acid, SOX10, and ferroptosis was investigated. To elucidate the functional role of boric acid in the SOX10/ferroptosis axis, U87 cells were treated with 5 μM Ferroportin‐1 (Fer‐1), a ferroptosis inhibitor, followed by stimulation with 532 μM boric acid for 24 h. Western blot and RT‐PCR analyses confirmed that boric acid downregulated SOX10 expression in U87 cells (Figure 3D–F). Specifically, boric acid reduced SOX10 protein and mRNA expression levels by 58.5% and 44.2%, respectively, compared to the control group (*p* < 0.0001). However, treatment with Fer‐1 significantly restored these levels to 69.4% and 72.5%, respectively (*p* < 0.0001).

**FIGURE 4:**
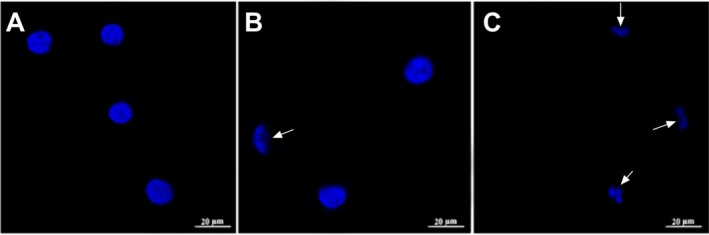
Nuclear morphological images stained with DAPI in U87 cells treated with boric acid and boric acid+Fer‐1. (A) Control group, (B) Boric acid+Fer‐1 treatment group, (C) Boric acid treatment group. White arrows indicate abnormalities in kidney‐shaped nuclei.

A similar trend was observed in the expression of GPx4 and ACSL4 at both the protein and mRNA levels (Figure 3D–F). In boric acid‐treated U87 cells, GPx4 protein and mRNA levels decreased by 64.6% and 51.8%, respectively, relative to the control (*p* < 0.0001), whereas ACSL4 protein and mRNA levels increased by 70.3% and 66.1%, respectively (*p* < 0.0001). Following Fer‐1 treatment, GPx4 expression and mRNA levels were upregulated, whereas ACSL4 expression and mRNA levels were downregulated, thereby counteracting the ferroptosis mechanism induced by boric acid. Additionally, the high expression of SOX10 in U87 cells was found to decline in response to boric acid‐induced ferroptosis.

Ferroptosis in boric acid‐treated U87 cells was evaluated through a series of experimental approaches. Consistent with the findings for GPx4 and ACSL4, Fe^2+^ (Figure 3G) and TFR levels (Figure 3D–F) were elevated following boric acid treatment. However, this effect was reversed by Fer‐1 treatment, which mitigated the increase in Fe^2+^ and TFR levels. Collectively, these findings indicate that boric acid induces ferroptosis in U87 cells by modulating Fe^2+^ and TFR levels, and altering the expression of ferroptosis‐related proteins such as GPx4 and ACSL4.

Furthermore, following a 24 h exposure to 532 μM boric acid, a significant reduction in cell density was observed, accompanied by the loss of typical cellular morphology (Figure 4A–C). The cells exhibited a rounded appearance, while the nuclei displayed irregular shapes (white arrows). These structural alterations suggest that boric acid may have disrupted the integrity of the microfilament cytoskeleton.

## 4 Discussion

Glioma, a notably invasive and aggressive brain tumour, continues to be difficult to treat due to the recurrent failure of current therapeutic strategies to prevent tumour relapse [26]. Extensive research has been undertaken to evaluate the anticancer properties of various potential therapeutic agents, with particular attention to their effects on critical signalling pathways such as cell cycle regulation, migration/invasion, apoptosis, ferritinophagy, and the HSPA5/NRF2/GPx4/GSH axis in glioblastomas [24, 25, 27, 28, 29]. In our previous study, we demonstrated that borax reduces glioblastoma cell viability by activating iron chaperones and autophagy signalling pathways [22]. Building on these findings, the present study investigated the impact of boric acid—a boron‐containing compound—on two distinct cell lines, U87 and HMC3. The objective was to assess its effects on glioma cells and determine the concentration required to induce cell death. The results provide compelling evidence that boric acid effectively promotes cell death in glioma cells by inducing ferroptosis, an iron‐dependent form of cell death characterised by the accumulation of lipid peroxides and an imbalance between prooxidants and antioxidants. Moreover, this study is the first to establish a link between SOX10 overexpression in glioma cells and its involvement in ferroptosis signalling, specifically through the GPX4/ACSL4 axis in U87 cells.

Previous studies have demonstrated the cytotoxic effects of boron compounds on glioblastoma cells. One study reported that boron concentrations of 2.5, 25, and 50 mM reduced the viability of T98G cells in a concentration‐dependent manner [30]. Similarly, Turkez et al. found that boric acid and borax (200–1000 μg/mL) significantly suppressed U87‐MG cell viability and exhibited anticancer properties by disrupting redox homeostasis, as determined by MTT analysis [31]. Additionally, another study investigated the cytotoxic effects of borax pentahydrate on U87‐MG cells at varying concentrations (ranging from 1 to 10,000 μM) and time points (24, 48, and 72 h) [32]. The results indicated that borax pentahydrate notably affected cell viability, particularly after 24 h, with an IC50 value of 2454 μM at 72 h. In the present study, the effects of boric acid on U87 and HMC3 cells were examined. CCK‐8 assay results revealed a concentration‐dependent reduction in U87 cell viability. Furthermore, boric acid treatment at concentrations ranging from 25 μM to 1.6 mM for 24 h exhibited antiproliferative effects in both U87 and HMC3 cells. Notably, the IC50 concentration required to reduce cell viability was higher in HMC3 cells (3.12 mM) than in U87‐MG cells (532 μM), indicating greater sensitivity of U87 cells to boric acid. Additionally, analysis revealed that U87 cells exhibited a greater sensitivity to boric acid than HMC3 cells at equivalent concentrations. BrdU incorporation analysis further confirmed a concentration‐dependent decrease in cell proliferation following boric acid treatment. Higher BrdU levels were detected in HMC3 cells compared to U87 cells, suggesting that boric acid induced more substantial cellular damage in U87 cells. Importantly, cell viability and proliferation analyses highlighted a key characteristic of boric acid—its relatively low cytotoxicity toward normal cells, or at least its reduced toxicity compared to its effects on cancer cells. Furthermore, variations in boron compound concentrations observed across different glioma studies, including this one, are likely attributable to differences in the specific boron compounds used, as well as variations in passage numbers among the cell lines analysed.

Previous studies have demonstrated that boron compounds exert cytotoxic effects on glioblastoma cells. For example, one investigation found that treating T98G cells with boron concentrations of 2.5, 25, and 50 mM led to a concentration‐dependent reduction in cell viability [30]. Similarly, Turkez et al. reported that both boric acid and borax, at concentrations ranging from 200 to 1000 μg/mL, significantly suppressed U87‐MG cell viability and disrupted redox homeostasis, as determined by MTT assays [31]. In another study, the cytotoxic effects of borax pentahydrate on U87‐MG cells were assessed over a concentration range of 1–10,000 μM at various time points (24, 48, and 72 h), with the results showing a pronounced reduction in cell viability—particularly after 24 h—and an IC50 of 2454 μM at 72 h [32]. In the present study, we examined the effects of boric acid on U87 and HMC3 cells. CCK‐8 assay results revealed a concentration‐dependent decrease in U87 cell viability. Additionally, treatment with boric acid at concentrations from 25 to 1.6 mM for 24 h exhibited antiproliferative effects in both cell lines. Notably, the IC50 for boric acid was 532 μM in U87 cells and 3.12 mM in HMC3 cells, indicating that U87 cells are more sensitive to boric acid. BrdU incorporation assays further confirmed a concentration‐dependent decline in cell proliferation, with higher BrdU levels observed in HMC3 cells compared to U87 cells, suggesting that U87 cells experienced more extensive cellular damage. Importantly, these findings underscore a key characteristic of boric acid—its relatively low cytotoxicity toward normal cells compared to its pronounced anticancer effects. Variations in effective concentrations reported across different studies are likely attributable to differences in the specific boron compounds used and variations in cell line passage numbers.

The SOX family was initially identified due to its highly conserved high‐mobility motifs, with early research primarily investigating its role in embryonic development and tissue differentiation [33]. However, subsequent studies have expanded this understanding, highlighting the involvement of SOX proteins in tumourigenesis as well. Several members of the SOX family have been implicated in cancer progression. Du et al. demonstrated that SOX13 upregulation enhances colorectal cancer metastasis by promoting the epithelial‐mesenchymal transition (EMT) process [34]. However, the role of SOX13 in treatment resistance, particularly in molecularly targeted therapies, remains largely unexplored. Yang et al. further revealed that SOX13 contributes to ferroptosis resistance, leading to therapy resistance in gastric cancer [35]. Given that ferroptosis‐mediated cell death plays a crucial role in enhancing the efficacy of chemotherapy and immunotherapy, identifying key regulators of ferroptosis may facilitate the discovery of novel therapeutic targets and biomarkers for monitoring treatment response. Similarly, SOX8 has been shown to exert potent ferroptosis‐promoting effects by reprogramming lipid, glucose, and iron metabolism, as well as modulating redox homeostasis [36]. Notably, SOX8‐induced ferroptosis is particularly effective against prostate cancer cells with high lipid demands [36]. Unlike other ferroptosis inducers, SOX8 does not require cystine deprivation and exhibits comparable lethality to the system Xc‐ inhibitor erastin. Additionally, Ikushima et al. showed that SOX4 is essential for maintaining the tumourigenic potential of glioma‐initiating cells through the upregulation of SOX2 [37]. Moreover, existing research underscores the clinicopathological and prognostic significance of SOX1 expression. High SOX1 expression has been identified as a negative prognostic biomarker in cancer, with reduced SOX1 protein and/or mRNA levels correlating with poorer prognosis and shorter overall survival in ovarian cancer, hepatocellular carcinoma, and oesophageal squamous cell carcinoma [38, 39, 40]. SOX9, a transcription factor critical for stem cell maintenance and plasticity, has also been implicated in glioma progression [41]. Previous studies by Aldaz et al. reported high SOX9 expression in glioma stem cells (GSCs), a finding supported by subsequent glioma research [42]. Functional analyses have demonstrated that SOX9 plays a key role in sustaining glioma stemness, further reinforcing its significance in tumour progression. In gliomas, the diverse members of the SOX family play essential roles in cell differentiation, and their mRNA expression levels have been associated with patient prognosis [15]. More recently, SOX10 expression profiles in gliomas have been analysed, revealing its potential as a prognostic marker [43]. Notably, SOX10 overexpression has been linked to poorer overall survival in glioblastoma patients and unfavourable prognosis. Furthermore, SOX10 has emerged as a potential predictor of immunotherapy response and immune effector activity, underscoring its relevance in glioma treatment strategies. SOX10 has also been strongly associated with an increased risk of perineural invasion in gastric cancer. Its overexpression has been shown to facilitate neural invasion by cancer cells, thereby contributing to tumour progression [44]. Given this critical role, targeting SOX10 inhibition may represent a promising therapeutic strategy for cancer treatment. Consistent with the aforementioned studies, our analysis of SOX10 protein and mRNA levels revealed that SOX10 expression was significantly higher in U87 cells compared to HMC3 cells.

From a biochemical perspective, the depletion of GSH and GPx4 by ferroptosis inducers compromises antioxidant capacity, leading to an increase in ROS‐mediated oxidative stress and ultimately promoting ferroptotic cell death [45]. Ferroptosis is a key determinant of cancer cell susceptibility, driven by GPX4 depletion, the accumulation of redox‐active iron, and peroxidized PUFA [46]. Consequently, ferroptosis‐related genes that suppress GPx4 expression and enzymatic activity, elevate intracellular iron levels, and enhance PUFA peroxidation have been widely explored as potential therapeutic targets in cancer treatment [47]. ACSL4 plays a pivotal role in ferroptosis by facilitating lipid peroxidation substrate deposition. A reduction in ACSL4 expression mitigates lipid peroxide accumulation, thereby inhibiting ferroptosis [48]. Previous studies have indicated that ACSL4 upregulation exacerbates ferroptosis‐induced brain injury and neuroinflammation, whereas its suppression promotes neurological recovery [49]. Chen et al. demonstrated that in Hemin‐induced HT‐22 cells, GSH and GPX4 levels were reduced, while Fe^2+^, ROS, and ACSL4 levels were elevated, along with a concomitant decrease in SOX10 expression [50]. These findings suggest that ACSL4 upregulation and GPx4 downregulation counteract the inhibitory effects of SOX10 overexpression on ferroptosis in Hemin‐induced HT‐22 cells. Consistent with previous studies, our findings demonstrated that boric acid treatment led to a decrease in GSH levels, while MDA and ROS levels increased, accompanied by a reduction in U87 cell count. Additionally, GPx4 and SOX10 expressions were downregulated, whereas ACSL4 expression was upregulated following boric acid exposure. Furthermore, boric acid treatment influenced iron metabolism in U87 cells by increasing transferrin receptor (TFR) and Fe^2+^ levels. Collectively, these findings indicate that boric acid effectively regulated the ferroptosis/SOX10 axis in U87 cells. Conversely, these changes were reversed upon treatment with Fer‐1, a ferroptosis inhibitor. Overall, boric acid treatment effectively downregulated the elevated SOX10 expression in glioma cells and robustly induced ferroptosis activation, resulting in a significant reduction in cell viability.

In summary, our findings provide the first evidence of the relationship between SOX10 and ferroptosis in glioma cells. We demonstrate that boric acid modulates the SOX10/GPX4/ACSL4 axis, elucidating the involvement of SOX10 signalling in ferroptosis within glioma cells. However, our study was limited to cellular‐level experiments, and we were unable to perform SOX10 downregulation or overexpression analyses, as well as investigations of other upstream and downstream target genes. Additionally, the SOX10/GPX4/ACSL4 axis was not validated through in vivo experiments, representing another important limitation. Moving forward, we aim to address these gaps using advanced experimental models, which we believe will generate critical insights to overcome clinical challenges in glioma treatment.

## Author Contributions

**Guven Kilic:** funding acquisition (equal), methodology (equal), resources (equal). **Ceyhan Hacioglu:** conceptualization (equal), data curation (equal), formal analysis (equal), funding acquisition (equal), investigation (equal), methodology (equal), project administration (equal), resources (equal), software (equal), supervision (equal), validation (equal), visualization (equal), writing – original draft (equal), writing – review and editing (equal). **Cengiz Tuncer:** resources (equal). **Ezgi Kar:** resources (equal). **Fatih Kar:** resources (equal). **Ahmet Taskesen:** resources (equal). **Adem Kurtulus:** resources (equal). **Onder Ipek:** resources (equal). **Oben Devin Cetiner:** resources (equal). **Cigdem Erdin:** resources (equal).

## Conflicts of Interest

The authors declare no conflicts of interest.

## Data Availability

The dataset is generated from the corresponding author on reasonable request.

## References

[1] D. N. Louis , A. Perry , G. Reifenberger , et al., “The 2016 World Health Organization Classification of Tumors of the Central Nervous System: A Summary,” Acta Neuropathologica 131, no. 6 (2016): 803–820, 10.1007/s00401-016-1545-1. 27157931

[2] Q. T. Ostrom , N. Patil , G. Cioffi , K. Waite , C. Kruchko , and J. S. Barnholtz‐Sloan , “Corrigendum to: CBTRUS Statistical Report: Primary Brain and Other Central Nervous System Tumors Diagnosed in the United States in 2013–2017,” Neuro‐Oncology 24, no. 7 (2022): 1214, 10.1093/neuonc/noaa269. 33340329 PMC9248396

[3] Q. T. Ostrom , L. Bauchet , F. G. Davis , et al., “The Epidemiology of Glioma in Adults: A “State of the Science” Review,” Neuro‐Oncology 16, no. 7 (2014): 896–913, 10.1093/neuonc/nou087. 24842956 PMC4057143

[4] R. Stupp , W. P. Mason , M. J. van den Bent , et al., “Radiotherapy Plus Concomitant and Adjuvant Temozolomide for Glioblastoma,” New England Journal of Medicine 352, no. 10 (2005): 987–996, 10.1056/NEJMoa043330. 15758009

[5] R. G. Verhaak , K. A. Hoadley , E. Purdom , et al., “Integrated Genomic Analysis Identifies Clinically Relevant Subtypes of Glioblastoma Characterized by Abnormalities in PDGFRA, IDH1, EGFR, and NF1,” Cancer Cell 17, no. 1 (2010): 98–110, 10.1016/j.ccr.2009.12.020. 20129251 PMC2818769

[6] A. P. Patel , I. Tirosh , J. J. Trombetta , et al., “Single‐Cell RNA‐Seq Highlights Intratumoral Heterogeneity in Primary Glioblastoma,” Science 344, no. 6190 (2014): 1396–1401, 10.1126/science.1254257. 24925914 PMC4123637

[7] V. Lefebvre , “Roles and Regulation of SOX Transcription Factors in Skeletogenesis,” Current Topics in Developmental Biology 133 (2019): 171–193, 10.1016/bs.ctdb.2019.01.007. 30902252 PMC6955022

[8] Y. Zhang and L. Hou , “Alternate Roles of sox Transcription Factors Beyond Transcription Initiation,” International Journal of Molecular Sciences 22, no. 11 (2021): 5949, 10.3390/ijms22115949. 34073089 PMC8198692

[9] D. Grimm , J. Bauer , P. Wise , et al., “The Role of SOX Family Members in Solid Tumours and Metastasis,” Seminars in Cancer Biology 67, no. Pt 1 (2020): 122–153, 10.1016/j.semcancer.2019.03.004. 30914279

[10] Y. Liu and W. Guo , “SOX Factors as Cell‐State Regulators in the Mammary Gland and Breast Cancer,” Seminars in Cell & Developmental Biology 114 (2021): 126–133, 10.1016/j.semcdb.2021.01.002. 33583737 PMC8154634

[11] I. Garcia , J. Aldaregia , J. Marjanovic Vicentic , et al., “Oncogenic Activity of SOX1 in Glioblastoma,” Scientific Reports 7 (2017): 46575, 10.1038/srep46575. 28425506 PMC5397861

[12] Y. Ge , F. Zhou , H. Chen , et al., “Sox2 Is Translationally Activated by Eukaryotic Initiation Factor 4E in Human Glioma‐Initiating Cells,” Biochemical and Biophysical Research Communications 397, no. 4 (2010): 711–717, 10.1016/j.bbrc.2010.06.015. 20537983

[13] T. Zhao , H. Yang , Y. Tian , et al., “SOX7 Is Associated With the Suppression of Human Glioma by HMG‐Box Dependent Regulation of Wnt/β‐Catenin Signaling,” Cancer Letters 375, no. 1 (2016): 100–107, 10.1016/j.canlet.2016.02.044. 26944317

[14] S. M. Glasgow , W. Zhu , C. C. Stolt , et al., “Mutual Antagonism Between Sox10 and NFIA Regulates Diversification of Glial Lineages and Glioma Subtypes,” Nature Neuroscience 17, no. 10 (2014): 1322–1329, 10.1038/nn.3790. 25151262 PMC4313923

[15] M. Stevanovic , N. Kovacevic‐Grujicic , M. Mojsin , M. Milivojevic , and D. Drakulic , “SOX Transcription Factors and Glioma Stem Cells: Choosing Between Stemness and Differentiation,” World Journal of Stem Cells 13, no. 10 (2021): 1417–1445, 10.4252/wjsc.v13.i10.1417. 34786152 PMC8567447

[16] M. Weider and M. Wegner , “SoxE Factors: Transcriptional Regulators of Neural Differentiation and Nervous System Development,” Seminars in Cell & Developmental Biology 63 (2017): 35–42, 10.1016/j.semcdb.2016.08.013. 27552919

[17] S. J. Dixon , K. M. Lemberg , M. R. Lamprecht , et al., “Ferroptosis: An Iron‐Dependent Form of Nonapoptotic Cell Death,” Cell 149, no. 5 (2012): 1060–1072, 10.1016/j.cell.2012.03.042. 22632970 PMC3367386

[18] J. Zheng and M. Conrad , “The Metabolic Underpinnings of Ferroptosis,” Cell Metabolism 32, no. 6 (2020): 920–937, 10.1016/j.cmet.2020.10.011. 33217331

[19] B. R. Stockwell , J. P. Friedmann Angeli , H. Bayir , et al., “Ferroptosis: A Regulated Cell Death Nexus Linking Metabolism, Redox Biology, and Disease,” Cell 171, no. 2 (2017): 273–285, 10.1016/j.cell.2017.09.021. 28985560 PMC5685180

[20] S. Doll , B. Proneth , Y. Y. Tyurina , et al., “ACSL4 Dictates Ferroptosis Sensitivity by Shaping Cellular Lipid Composition,” Nature Chemical Biology 13, no. 1 (2017): 91–98, 10.1038/nchembio.2239. 27842070 PMC5610546

[21] J. P. Friedmann Angeli , D. V. Krysko , and M. Conrad , “Ferroptosis at the Crossroads of Cancer‐Acquired Drug Resistance and Immune Evasion,” Nature Reviews. Cancer 19, no. 7 (2019): 405–414, 10.1038/s41568-019-0149-1. 31101865

[22] C. Hacioglu and C. Tuncer , “Boric Acid Increases Susceptibility to Chemotherapy by Targeting the Ferritinophagy Signaling Pathway in TMZ Resistant Glioblastoma Cells,” Biological Trace Element Research 202, no. 8 (2024): 3574–3587, 10.1007/s12011-023-03930-7. 37906374

[23] S. Shimizu , K. Nakai , Y. Li , et al., “Boron Neutron Capture Therapy for Recurrent Glioblastoma Multiforme: Imaging Evaluation of a Case With Long‐Term Local Control and Survival,” Cureus 15, no. 1 (2023): e33898, 10.7759/cureus.33898. 36819302 PMC9937644

[24] C. Tuncer and C. Hacioglu , “Borax Induces Ferroptosis of Glioblastoma by Targeting HSPA5/NRF2/GPx4/GSH Pathways,” Journal of Cellular and Molecular Medicine 28, no. 7 (2024): e18206, 10.1111/jcmm.18206. 38494858 PMC10945083

[25] C. Hacioglu , F. Kar , F. Davran , and C. Tuncer , “Borax Regulates Iron Chaperone‐ and Autophagy‐Mediated Ferroptosis Pathway in Glioblastoma Cells,” Environmental Toxicology 38, no. 7 (2023): 1690–1701, 10.1002/tox.23797. 36988300

[26] F. Hanif , K. Muzaffar , K. Perveen , S. M. Malhi , and S. U. Simjee , “Glioblastoma Multiforme: A Review of Its Epidemiology and Pathogenesis Through Clinical Presentation and Treatment,” Asian Pacific Journal of Cancer Prevention 18, no. 1 (2017): 3–9, 10.22034/APJCP.2017.18.1.3. 28239999 PMC5563115

[27] Y. C. Chou , M. Y. Chang , M. J. Wang , et al., “Phenethyl Isothiocyanate Alters the Gene Expression and the Levels of Protein Associated With Cell Cycle Regulation in Human Glioblastoma GBM 8401 Cells,” Environmental Toxicology 32, no. 1 (2017): 176–187, 10.1002/tox.22224. 26678675

[28] Y. Y. Chen , Y. M. Chang , K. Y. Wang , et al., “Naringenin Inhibited Migration and Invasion of Glioblastoma Cells Through Multiple Mechanisms,” Environmental Toxicology 34, no. 3 (2019): 233–239, 10.1002/tox.22677. 30431227

[29] H. S. Shang , Y. L. Shih , T. J. Lu , et al., “Benzyl Isothiocyanate (BITC) Induces Apoptosis of GBM 8401 Human BRAIN Glioblastoma Multiforms Cells via Activation of Caspase‐8/Bid and the Reactive Oxygen Species‐Dependent Mitochondrial Pathway: BITC INDUCE APOPTOSIS IN GBM 8401 HUMAN BRAIN GLIOBLASTOMA MULTIFORMS CELLS,” Environmental Toxicology 31, no. 12 (2016): 1751–1760, 10.1002/tox.22177. 28675694

[30] H. E. Aydin , M. K. Gunduz , C. Kizmazoglu , T. Kandemir , and A. Arslantas , “Cytotoxic Effect of Boron Application on Glioblastoma Cells,” Turkish Neurosurgery 31, no. 2 (2020): 206–210, 10.5137/1019-5149.JTN.30316-20.1. 33372254

[31] H. Turkez , M. E. Arslan , A. Tatar , and A. Mardinoglu , “Promising Potential of Boron Compounds Against Glioblastoma: In Vitro Antioxidant, Anti‐Inflammatory and Anticancer Studies,” Neurochemistry International 149 (2021): 105137, 10.1016/j.neuint.2021.105137. 34293392

[32] B. Çelik , E. Ersöz , and M. Korkmaz , “Boraks Pentahidrat'ın Glioblastoma Multiforme Hücre Hattındaki Tedavi Potansiyelinin Aras¸Tırılması,” Journal of Boron 5, no. 1 (2020): 56–61, 10.30728/boron.589644.

[33] V. Lefebvre , “The SoxD Transcription Factors—Sox5, Sox6, and Sox13—Are Key Cell Fate Modulators,” International Journal of Biochemistry & Cell Biology 42, no. 3 (2010): 429–432, 10.1016/j.biocel.2009.07.016. 19647094 PMC2826538

[34] F. Du , X. Li , W. Feng , et al., “SOX13 Promotes Colorectal Cancer Metastasis by Transactivating SNAI2 and c‐MET,” Oncogene 39, no. 17 (2020): 3522–3540, 10.1038/s41388-020-1233-4. 32111984

[35] H. Yang , Q. Li , X. Chen , et al., “Targeting SOX13 Inhibits Assembly of Respiratory Chain Supercomplexes to Overcome Ferroptosis Resistance in Gastric Cancer,” Nature Communications 15, no. 1 (2024): 4296, 10.1038/s41467-024-48307-z. PMC11106335 38769295

[36] X. Yang , C. Gu , J. Cai , et al., “Excessive SOX8 Reprograms Energy and Iron Metabolism to Prime Hepatocellular Carcinoma for Ferroptosis,” Redox Biology 69 (2024): 103002, 10.1016/j.redox.2023.103002. 38142583 PMC10788634

[37] H. Ikushima , T. Todo , Y. Ino , M. Takahashi , K. Miyazawa , and K. Miyazono , “Autocrine TGF‐β Signaling Maintains Tumorigenicity of Glioma‐Initiating Cells Through Sry‐Related HMG‐Box Factors,” Cell Stem Cell 5, no. 5 (2009): 504–514, 10.1016/j.stem.2009.08.018. 19896441

[38] H. Y. Su , H. C. Lai , Y. W. Lin , Y. C. Chou , C. Y. Liu , and M. H. Yu , “An Epigenetic Marker Panel for Screening and Prognostic Prediction of Ovarian Cancer,” International Journal of Cancer 124, no. 2 (2009): 387–393, 10.1002/ijc.23957. 18942711

[39] J. Lou , K. Zhang , J. Chen , Y. Gao , R. Wang , and L. B. Chen , “Prognostic Significance of SOX‐1 Expression in Human Hepatocelluar Cancer,” International Journal of Clinical and Experimental Pathology 8, no. 5 (2015): 5411–5418. 26191244 PMC4503115

[40] A. Rad , S. Esmaeili Dizghandi , M. R. Abbaszadegan , N. Taghechian , M. Najafi , and M. M. Forghanifard , “SOX1 Is Correlated to Stemness State Regulator SALL4 Through Progression and Invasiveness of Esophageal Squamous Cell Carcinoma,” Gene 594, no. 2 (2016): 171–175, 10.1016/j.gene.2016.08.045. 27576349

[41] Z. Wang , X. Xu , N. Liu , et al., “SOX9‐PDK1 Axis Is Essential for Glioma Stem Cell Self‐Renewal and Temozolomide Resistance,” Oncotarget 9, no. 1 (2017): 192–204, 10.18632/oncotarget.22773. 29416606 PMC5787456

[42] P. Aldaz , N. Martín‐Martín , A. Saenz‐Antoñanzas , et al., “High SOX9 Maintains Glioma Stem Cell Activity Through a Regulatory Loop Involving STAT3 and PML,” International Journal of Molecular Sciences 23, no. 9 (2022): 4511, 10.3390/ijms23094511. 35562901 PMC9104987

[43] G. Xiao , K. Wang , Z. Wang , et al., “Machine Learning‐Based Identification of SOX10 as an Immune Regulator of Macrophage in Gliomas,” Frontiers in Immunology 13 (2022): 1007461, 10.3389/fimmu.2022.1007461. 36524115 PMC9745112

[44] S. A. M. Yazdi , A. Moghtadaie , and E. Nazar , “The Value of SOX10 Expression in Predicting Perineural Invasion in Gastric Cancer,” Revista Española de Patología 56, no. 4 (2023): 227–232, 10.1016/j.patol.2023.05.003. 37879819

[45] J. Li , F. Cao , H. L. Yin , et al., “Ferroptosis: Past, Present and Future,” Cell Death & Disease 11, no. 2 (2020): 88, 10.1038/s41419-020-2298-2. 32015325 PMC6997353

[46] D. Tang , X. Chen , R. Kang , and G. Kroemer , “Ferroptosis: Molecular Mechanisms and Health Implications,” Cell Research 31, no. 2 (2021): 107–125, 10.1038/s41422-020-00441-1. 33268902 PMC8026611

[47] H. Wang , Y. Cheng , C. Mao , et al., “Emerging Mechanisms and Targeted Therapy of Ferroptosis in Cancer,” Molecular Therapy 29, no. 7 (2021): 2185–2208, 10.1016/j.ymthe.2021.03.022. 33794363 PMC8261167

[48] L. E. Pope and S. J. Dixon , “Regulation of Ferroptosis by Lipid Metabolism,” Trends in Cell Biology 33, no. 12 (2023): 1077–1087, 10.1016/j.tcb.2023.05.003. 37407304 PMC10733748

[49] Y. Cui , Y. Zhang , X. Zhao , et al., “ACSL4 Exacerbates Ischemic Stroke by Promoting Ferroptosis‐Induced Brain Injury and Neuroinflammation,” Brain, Behavior, and Immunity 93 (2021): 312–321, 10.1016/j.bbi.2021.01.003. 33444733

[50] H. Chen , L. Ren , and W. Ma , “Mechanism of SOX10 in Ferroptosis of Hippocampal Neurons After Intracerebral Hemorrhage via the miR‐29a‐3p/ACSL4 Axis,” Journal of Neurophysiology 129, no. 4 (2023): 862–871, 10.1152/jn.00374.2022. 36919939

